# Therapeutic aphorisms for paranoid personality disorder and paranoid ideation treatment: psychological opposites and complementaries in brief therapy

**DOI:** 10.3389/fpsyg.2023.1278721

**Published:** 2024-01-16

**Authors:** Bernardo Paoli

**Affiliations:** Ordine degli Psicologi del Lazio, Rome, Italy

**Keywords:** aphorisms, brief therapy, paranoid personality disorder, proverbs, psychological opposites and complementaries, therapeutic communication

## Abstract

**Background:**

In brief therapy (BT), aphorisms (As) have been considered a privileged element in therapeutic language. With a handful of words, they arouse emotions and different perspectives.

**Theoretical proposal:**

In As, a specific structure—the implicit and explicit symmetry of opposites (SoO)—allows a surprise effect, which in turn allows a therapeutic effect. Therefore, a carefully selected SoO (based on psychological complementaries) underlies the desired therapeutic reframing, which can be identified as a core goal of the psychotherapeutic intervention.

**Research question:**

Is it possible to identify As with therapeutic value in treating paranoid personality disorder (PPD) and high paranoid ideation (hPI) cases?

**Method:**

Starting from an initial collection of 369 original As created on the spot in 2,335 BT sessions, As used during on-line psychotherapy on 55 patients with PPD and/or an hPI measured with the SCL-90-R were identified.

**Results:**

Thirty-three As were selected and organised according to effective and ineffective strategies, the target of the BT intervention for PPD and hPI cases.

*“An aphorism never coincides with the truth:*

*it is either a half-truth or one-and-a-half truths.”*

Karl Kraus (1972)

## Paroemias: history and goals of proverbs and aphorisms

Paroemias are the most ancient linguistic form of science and knowledge transfer: short, sharp, memorable quotes handed down in written or oral form with an ethical and sapiential content, useful for instructing people in the art of living. The term “paroemia” refers to a broad spectrum of linguistic phenomena, including proverbs (Ps) and aphorisms (As) as well. Saying a lot in a nutshell (Franceschini, 2007): a paroemia is a communication tool made of short, wise quotes (Mieder, 1993) whose conciseness is inversely proportional to their deepness (Levergeois, 2006), briefly expressing truths drawn from experience (Lapucci, 2007). The greater the wit paroemias are expressed with—and the denser their information is—the greater, *ipso facto*, is their ability to convey knowledge (Poccetti, 1989). They are the little things helping the big ones thrive (Limentani, 2006), a happy briefness (Rigoni, 2006). They belong to the same genre as the treatise and the essay, but, due to their extremely concise nature, they show a lack of argumentation, thus becoming sententious quotes (Helmich, 2006). As for the difference between Ps and As, if the former belong primarily to the oral and community tradition, the latter belong to the written tradition and express an individual thought; Ps are ascribable to a popular tradition, while As refers to a learned tradition (Cristilli, 1989). In the first case, authoritativeness relies on a collective basis and resides in the communal wisdom of a population; in the second case, however, skills and social position granted to the individual author make the A authoritative.

The P boasts an important and ancient history, and it can be found in all cultures and in all ages, with rare exceptions; the Sumerians provided us with the oldest written evidence of Ps (18th century B.C.) (Lapucci, 2007). Sumerian Ps are surprising for the topicality of their themes, despite the differences within society, customs, economy, and religion. An example: “Who possesses much silver may be happy; who possesses much barley may be glad; but he who has nothing at all may sleep” (Lapucci, 2007). Even among the ancient Egyptians, the P was of remarkable importance. Initially closely linked to religious morality, it diverged from it to evolve into a set of practical and behavioural advice and rules; an example dating back to mid-second-millennium B.C.: “Bread given with love is more valued than a capon given with resentment” (Lapucci, 2007). The much better-known collection of ancient Jewish wisdom, the *Proverbs* (Vattioni, 1998), is directly influenced by Egyptian wisdom. The coincidences between some biblical Ps and, for example, some maxims from the *Instruction of Amenemope*—developed in the world of the Pharaohs and containing advice on the art of living, succeeding in life, and being happy—have long been pointed out (Cimosa, 2007). The *Proverbs*, too, have a clear educational purpose: they aim to humanistically train young people to let them discover the meaning of life and acquire prudence, affability, patience, self-control, and optimism (Cimosa, 2007).

Even in ancient Greece, sententious quotes played a central role (see Lelli, 2021). For example, the very first written document of Hellenicity that has come down to us—the “Nestor’s cup” (8th century BC)—offers a sententious evidence of one of the most widespread Ps in ancient and modern Western tradition, centred on the dangers of wine and love and their dire consequences for mankind. Nevertheless, the Seven Sages (7th–6th century B.C.) are the first real protagonists of the Greek sententious culture, condensing their thoughts into short, often sharp quotes. An example: “Do not rush when attending friends’ dinners, but hasten to their misfortunes” by Chilon of Sparta (Lelli, 2021).

As hold a special place in the history of medicine. The word “aphorism” was first used by Hippocrates of Kos (460 - c. 370 B.C.), regarded as the father of scientific medicine. He used this term as the title of his collection of sayings, in which he set out some medical principles (Hippocrates, 1994). During the 19th, 20th, and 21st centuries, several authors in the medical field developed As related to neuroscience and neurorehabilitation that helped to improve our understanding of the nervous system and, therefore, the management of the neurological patient (Cano-de-la-Cuerda, 2021). Medical As are recognised as educating for narrative sensitivity, as a help to memorise instructions, to inform the patient about clinical judgement, to reinforce the physician’s professional behaviour, and to build the physician’s professional identity (Levine and Bleakley, 2012). For example, the A: “It looks like this, but what else could it be?” (Croskerry, 2009) can be a useful reminder to avoid premature closure in diagnosis.

The short forms deriving from the Greek tradition can be considered the basis of the literary genre of the A; over the centuries, the evolution of the sententious material has been increasingly driven by the need to ascribe the sayings to a specific author (Tosi, 2006).

Notwithstanding the long gnomic tradition mentioned—and the historical precedent of Baltasar Gracián’s *Oráculo manual y arte de prudencia* dated 1647 (Gracián, 2020)—the linguistic object called “aphorism” by modern literary theorists appeared in 1664 with the first edition of François de La Rochefoucauld’s *Réflexions ou sentences et maximes morales* (de La Rochefoucauld, 1993). La Rochefoucauld established a literary expression with a subjective and intimist nature, intended for a generalist audience (Helmich, 2006). A famous example: “Our virtues are most frequently but vices in disguise.”

## Aphorism structure and two theoretical proposals

What are the differences between As/Ps and other linguistic forms? (see Lambertini, 2022). As and Ps consist of (a) at least one sentence; (b) stating a general, experiential, or theoretical truth; (c) on the human being; (d) being self-sufficient, i.e., they can be isolated from the rest of the text. Moreover, two other components seem to specifically distinguish As (see Adorno, 2006): (e) the binary/antinomic composition, (f) and the wit/surprise effect.

From 1933 onwards, the *pointe* (Helmich, 2006) has played an essential structural role for As, highlighting the final effect the A should trigger in the reader/listener: an aesthetic and gnoseological surprise (Helmich, 1991) resulting in a more or less profound re-evaluation of personal beliefs, or in laughter, or in both (Helmich, 2006). More recently, the theme-rheme (or “topic-comment”) structure (Dundes, 1994; Eggins, 2004) has been unanimously recognised by the scientific community as a defining feature in Ps and As (Lambertini, 2022). They are both composed of at least one sentence and thus of at least one subject (which would correspond to the theme) and one predicate (which would correspond to the rheme) (Dundes, 1994; Lambertini, 2022); the theme-rheme pair talks about the message of a sentence and the progress of communication. For example, an Al-Hikam—A from the Sufi tradition—“The source of every disobedience, indifference, and passion is self-satisfaction. The source of every obedience, vigilance, and virtue is dissatisfaction with one’s self”: “The source of every disobedience, indifference, and passion” is the theme (old), “is self-satisfaction” is the rheme (new); “The source of every obedience, vigilance, and virtue” is the theme (old), and “is dissatisfaction with one’s self” is the rheme (new) (Anis, 2023).

In As—this is the former theoretical proposal of this paper—their binary structure becomes antinomic (a dialogue between opposites) that triggers an aesthetic and gnoseological surprise (surprise effect), which can determine an emotional and perspective shift (therapeutic reframing) (see Watzlawick, 1977). Those opposites producing said therapeutic change should acquire a specific status of “complementaries” because they are opposites working at a psychotherapeutic level (Paoli, 2019). As already highlighted, suggesting the opposite of what is already known produces a surprise effect (denying what is traditionally assumed—taken-for-granted assumptions of an imagined audience—makes a topic interesting; Davis, 1971); there is a close connection among antinomies, interesting/creative, and therapeutic reframing (Watzlawick et al., 1974; Watzlawick, 1977; Paoli, 2014); “doing the opposite” does not always involve a therapeutic/effective change and it is necessary to distinguish between psychological opposites (first-order change/apparent change) and psychological complementaries (second-order change/actual change) (Watzlawick et al., 1974; Paoli, 2019). In this study, the relationship among these themes is explored and applied to therapeutic As.

The latter proposal aims at identifying two possible types of antinomic structures in As: implicit and explicit. This proposal has been referred to as “implicit and explicit symmetry of opposites” (see Paoli, 2018, 2019) in order to bring the opposites (a topic present in philosophy since its dawn; Severino, 1996) back to the theme of symmetry, which also brings together art, shapes of nature, mathematical formulae, physical laws, and the ability of science to make predictions (Castellani, 2000; Ghirardi, 2019a,b). Among all the various forms of symmetries, the symmetry of opposites (SoO) represents just one of the possible cases.

**Figure 1:**
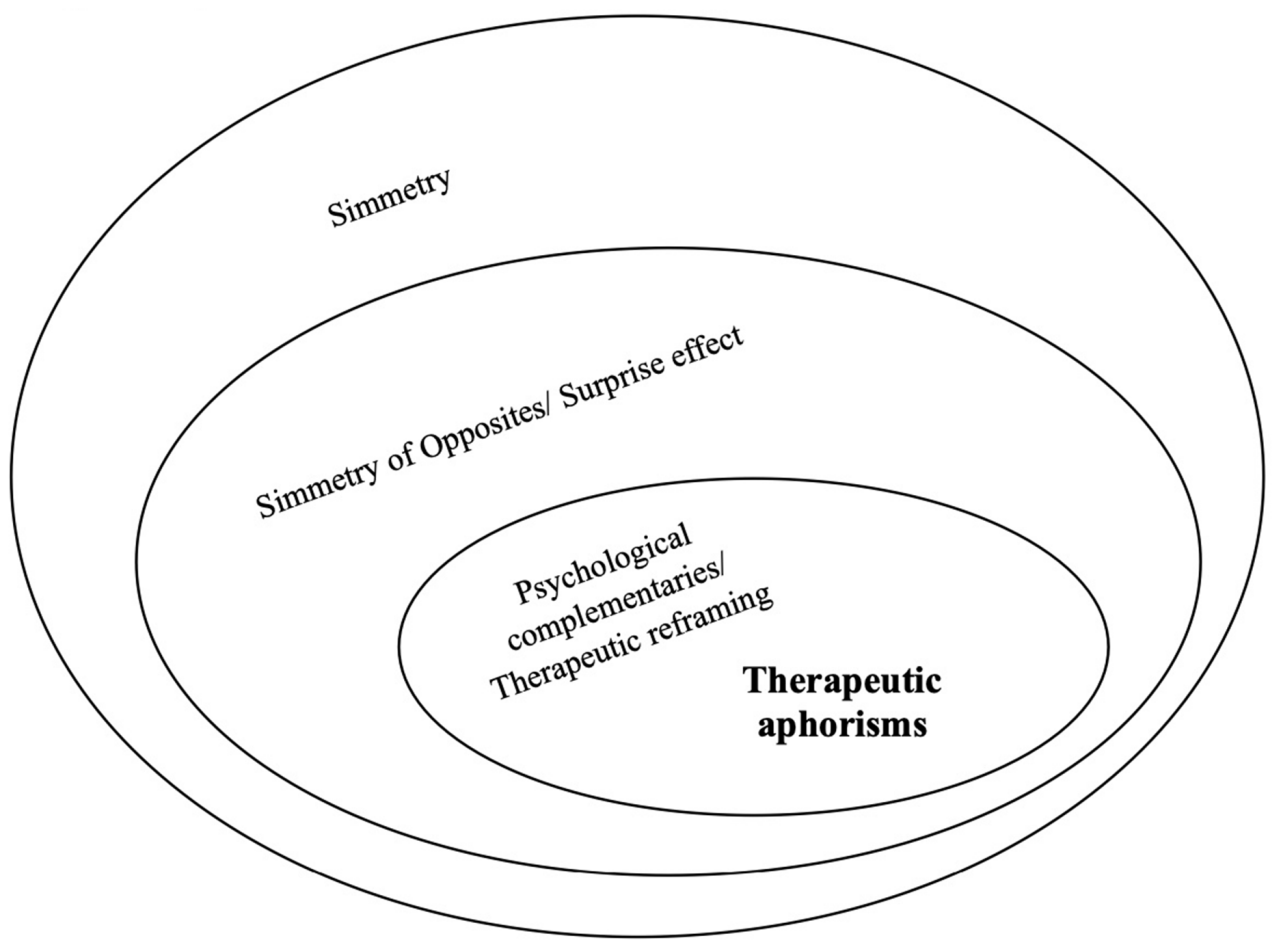
Therapeutic aphorisms and their relation toward symmetry, symmetry of opposites, and psychological complementaries.

To summarise the theoretical proposal of this paper, in As, a specific symmetrical structure—the implicit and explicit SoO—allows a surprise effect, which in turn allows a therapeutic effect. Although the surprise effect relies on a symmetrical structure, not all symmetrical structures generate a surprise effect; likewise, although the therapeutic ability of an A relies on the presence of a SoO, not all SoOs lead to a therapeutic effect (Figure 1). So, a carefully selected SoO in As allows the desired therapeutic reframing, also called “reappraisal” (Troy et al., 2013). This (possible) relation is the reason why As can be considered an extremely useful tool in psychotherapy; reframing can be identified as a core goal of the psychotherapeutic intervention (Watzlawick, 1977; Paoli, 2014).

## Implicit and explicit symmetry of opposites in aphorisms

The SoO in As can show either an implicit or an explicit form (Paoli, 2018). Frequently, the SoO is explicit; in this case, the A clearly shows two opposites put side by side, in an apparent contrast. An example: “The relationship only began to work when love was over” by Merini (2022). Merini opposes *beginning* and *being over* but, above all, *working* and *love*, thus painting—with a few brushstrokes—a typical human contradiction, whereby it is precisely the desire to make a relationship work at all costs, through love, that determines its impossibility of working.

On the other hand, in an implicit SoO, the A shows a contrasting point of view toward an opposite external to the A itself. It is a contrast between what is advocated by A and what corresponds to the common way of feeling the reader/listener implicitly refers to. As Merini wrote, “I am greater than any betrayal” (Merini, 2022). The contrast is against common sense, in which betrayal is an action suffered: something so tough and painful that it can make the person feel *small*. Merini suggests an opposite point of view: betrayal can be overcome, as long as the person recognises to be *greater* than any infidelity.

Moreover, a therapeutic A, even with an explicit SoO, may also contain an implicit SoO because it contrasts (and therefore reframes) the “common sense,” which can be a vehicle for imbalance. In the first example A, characterised by an explicit SoO, also contains an implicit one, implicitly opposing the common perception that love is enough to make a relationship work.

## Aphorisms and brief therapy

Brief therapy (BT) (Watzlawick et al., 1967, 1974; Haley, 1973, 1985a,b,c, 1987; Weakland et al., 1974; Watzlawick, 1977; Fisch et al., 1982; de Shazer, 1988; Gulotta, 1997; O’Hanlon and Beadle, 1997; Watzlawick and Nardone, 1997; Soo-Hoo, 1997, 2019; Fisch and Schlanger, 1999; Hoyt, 2009; Huibers, 2010; Hagg, 2011; Cepukiene and Pakrosnis, 2011; Petruccelli and Verrastro, 2012; Hoyt and Talmon, 2014; Paoli, 2014, 2019; Pietrabissa et al., 2016; Broadman, 2017; Cannistrà and Piccirilli, 2018; Kim et al., 2019; Solis and Vargas, 2019; Vitry et al., 2019, 2021; Koorankot, 2021; Proietti et al., 2022; Asai and Asai, 2023; Espugnatore et al., 2023) has intercepted the therapeutic value of As (Watzlawick, 1977; Nardone, 2007; Rampin, 2007; Paoli, 2014) to such an extent that in BT training, students are invited to practice the study of As. A brief examination of some of the founding principles of BT (referred to in the *Encyclopedia of Psychotherapy* as “Contextual Brief Therapies”; Steenbarger, 2002) will be useful to better understand the reason behind the predilection of this therapeutic approach toward the As:

1. BT is a goal-oriented, efficiency-centered, interaction and resource-based, balance-experience-focused psychotherapy.
2. Patients/clients - and people/environments around them - have plenty of resources that can be leveraged for change.
3. Psychological disorders can be understood as problems to be solved, and the process of change as a process of goal-setting and problem-solving.
4. Human problems, or the temporary impossibility of achieving a goal, can be described as feedback loops (cybernetic loops) that correspond to homeostasis in interactional systems characterised by circular causality (interaction within people themselves and between people within couples, families, groups, communities, and societies). These feedback loops are dependent, fed and complicated by ineffective strategies (ISs) taken in an attempt to manage the situation (the “attempted solutions”).
5. The solution of a human problem and the formulation of a new feedback loop corresponding to a new homeostasis depend on the abandonment of the ISs and on the use of effective strategies (ESs). Regarding the situation perceived as a problem, ESs are often already present; for this reason they are also called “positive exceptions to the problem” and they are considered resources to be leveraged. ESs have to be strengthened and amplified.
6. ISs and ESs are linguistic, physical, and mental acts used deliberately or not, consciously or unconsciously, by one or more people. They are symmetrically opposed: ISs rotated by 180 degrees become ESs, and vice versa.
7. There is a distinction between opposites and complementaries: not all 180-degree rotated strategies (opposite strategies) work; complementary strategies are opposite strategies that are effective. A distinction has to be made between first-order and second-order change: a first-order change is just an apparent change because opposite-not-complementary strategies are used; a second-order change is an actual change produced through complementary strategies.
8. Brief therapists design/co-construct balance experiences (therapeutic experiences) with their patients/clients based on ESs (complementary strategies).

Therapeutic As reflect the distinction between opposites and complementaries: only those built on psychological complementaries are effective and also meaningful (see Paoli et al., 2022; Paoli, 2022a,b). In therapeutic As, the SoOs—both implicit and explicit—are selected in such a way that the points of view of the As are complementary to the typical ISs of the specific problems/disorders to be treated, and they are adherent to the typical ESs that allow the problems/disorders to be solved and the goals to be reached. Therapeutic As arouse aversion toward the ISs and they promote adherence toward the ESs (see Table 1).

**Table 1:** Main features of situation-problem and situation-solution.

Problem	Solution
Ineffective strategies (attempted solutions and opposite-not-complementary strategies).	Effective strategies (exceptions to the problem and complementary strategies).
First-order change (apparent change).	Second-order change (actual change).
Psychological opposites (ineffective opposites).	Psychological complementaries (effective opposites).	Imbalance experiences (psychopathogenic experiences).	Balance experiences (therapeutic experiences).
Non-utilization of resources.	Utilization of resources.
Decrease in possibilities.	Increase in possibilities.	Absence of meaningfulness.	Presence of meaningfulness.
Aphorisms that arouse aversion toward effective strategies.	Aphorisms that arouse adherence toward effective strategies.
Aphorisms that arouse adherence toward ineffective strategies.	Aphorisms that arouse aversion toward ineffective strategies.

A psychotherapy session can involve the use of famous As, or psychotherapists themselves can devise short sentences with the same features as an A. In the latter case, the authoritativeness of the As does not depend on the name of the famous author quoted but on the asymmetry that defines the therapeutic relationship.

## Research question and method: aphorisms for treating paranoid personality disorder and high paranoid ideation cases

The leading research question was: Is it possible to identify As with therapeutic value in treating paranoid personality disorder (PPD) and high paranoid ideation (hPI) cases?

In the first step, all As created on the spot and used during 2,335 BT sessions were collected. As were identified thanks to the reaction of patients, who emphasised—during the session or during subsequent sessions—that those words had been useful to them. The collection includes 369 original As, freely accessible (in Italian) at this link: https://bernardopaoli.it/cosa-sono-gli-aforismi/, which represented the database for therapeutic As.

In a second research phase, a sample of 213 patients was taken into consideration; all patients who applied for an online BT within 24 months were included in the sample. At the beginning of the therapy, 7.98% (N 17) matched the diagnosis of PPD (Black and Grant, 2015), and 22.07% (N 47) reported a high value in the Paranoid Ideation Index in the SCL-90-R test (a value higher than 65 out of 75). The final target observation sample consisted of a total of 55 patients with PPD and/or hPI (9 patients both matched the diagnosis of PPD and had a hPI). The online BT of the 55 patients lasted up to 367 sessions in total, an average of 6.67 sessions per patient.

The criteria for the selection of the As were: being among the 369 As in the initial database; and being used during the 367 target sessions. Thirty-three As were identified and then categorised according to the ISs (toward which arouse aversion) and the ESs (toward which arouse adherence) on which the work in BT is focused.

## Psychological complementaries and (in)effective strategies in paranoid personality disorder and high paranoid ideation

Patients with PPD (Black and Grant, 2015; Frattura et al., 2019; Lingiardi and McWilliams, 2020) tend to show an attitude of demand toward others; they expect people to respect them and to be well-liked. Demand, however, is a relational repellent. When looking for an alternative, opposite relational strategy, some patients move from demand to relinquishment, or from demand to showing emotional neediness. In both cases, these are ISs: surrendering is the gateway to depression, and showing an emotionally needy behaviour burdens the relationship by putting the patient in a position of annoying passivity. What is the complementary strategy, the effective opposite? The complementary of demand is seductiveness, highlighting personal seductive aspects (Paoli, 2019); a complementary that can be communicated to the patient with an A like this: “If a good relationship is what you are looking for, start attracting others rather than mistreating them.” Similarly, with anger (the ineffective management of which is a typical topic in PPD cases) its complementary is not calmness but strength. In fact, if an angry person is urged to calm down, this request has a reverse effect: the person will get even angrier. A different message creates a different result: “Every time you explode with anger you show your weakness and you can be controlled easily: as soon as someone tells something to you, you flare up. A strong person does not act with rage.”

A number of ISs and ESs have been identified in BT to work with PPD and paranoid ideation (see Muriana and Verbitz, 2017; Paoli, 2020) which can be summarised as follows:

### Main ineffective strategies

(1) Dwelling on angry thoughts. (2) Complaining to and about others, about the lack of ethics. (3) Expressing their thoughts without filters. (4) Distrusting people and people’s intentions (including their own partner, toward whom they feel a lot of jealousy), and believing that aggressively communicating their own anger is an expression of strength. (5) Getting defensive and saying no to others’ proposals. (6) Imagining and perceiving a world full of enemies and obstacles. (7) Devaluing the importance of seduction and of being seductive; expecting (or even demanding) that people they feel attraction toward are interested in turn. (8) Believing that their ethics are unparalleled and that other people are disappointing. (9) Spending time with people and in environments they deem toxic, expecting those environments and people to change and adapt to them and their ethics. (10) Giving to people toward whom they feel predilection more than is required by others and the environment, expecting gratitude in return.

### Main effective strategies

(1) Writing angry letters (which are not meant to be sent to the recipient). (2) Taking time—writing on their own or talking to a friend who listens without replying—to deliberately complain about everything that is wrong in their own lives and in the behaviour of other people. (3) Improving the effectiveness of their communication, also taking into consideration the use of shrewdness in order to achieve specific and substantial goals. (4) Believing that aggressively communicating their anger is inappropriate; showing kindness toward other people. (5) Exercising trust toward others strategically as a (pleasant) form of evaluating other people’s intentions; saying yes to others’ proposals. (6) Imaging and perceiving a world full of friends and opportunities. (7) Acknowledging and indulging in the importance that natural evolution has granted to seduction and courtship. (8) Believing that everyone is entitled to their own ethics and that it is quite normal for human beings—patients included—to act primarily according to the logic of a personal benefit; actively contributing to the achievement of others’ benefits. (9) Completely cutting out or reducing the time spent with people and in environments considered toxic, devoting more time to fulfilling and meaningful relationships. (10) Acting with an attitude of real gratuitousness.

## Results: a selection of 33 aphorisms for patients with paranoid personality disorder and high paranoid ideation

Below are the 33 As, selected and organised according to the previously mentioned criteria.

*(A) Arousing aversion toward:*

*(A1) Unfiltered expression of anger.*

1. The opposite of anger is not calmness, but strength.

2. Anger hides the fear of losing something important.

3. People who get angry believe to be valuable.

4. Anger always shows three signals. First: you get angry because the matter is worth it. Second: you must perform an action. And third: it’s never the first one that you thought of.

5. Anger is a good motivator, but a bad counsellor.

*(A2) Complaining to others, demanding and perceived disappointment toward others.*

6. Whoever complains always does it to the wrong person.

7. Every complaint hides a desire.

8. Complaining is a concession of power.

9. People who always complain see the world as their personal toilet.

10. Demand is the strongest repellent in the world: it drives the best people away from you.

11. When you say that you do not care anymore about someone, it means you still care a lot.

12. When people let you down they are showing you what they are like and not what you guessed they were like.

*(A3) Negative evaluation of other people’s intentions, jealousy toward the partner and paranoid ideation.*

13. Jealousy is paranoia disguised as love.

14. A jealous person feels uninteresting.

15. A paranoid sees well, but way too much.

16. When you only see enemies around you it means that the enemy is within you.

*(B) Arousing adherence toward:*

*(B1) A positive outlook and/or a positive reaction toward others.*

17. You will always meet people who will try to stoop to their level. Not out of malice, but because it is the only one they know.

18. When people around you try to make you feel guilty about being single, show them your happiness.

19. The best way to evaluate yourself is asking other people.

20. The rule is that if you isolate yourself, the world isolates you; if you get involved, the world will get you involved.

*(B2) Openness toward others: attitude of gratuitousness and benefits for others.*

21. Allow yourself to do only what you are able to do gratuitously.

22. When you struggle to receive, it is because you struggle to give.

23. When meeting other people always ask yourself: ‘How can I bring them some personal benefit?’

*(B3) Seeking a healthy proximity/distance and a good selection of the environment to spend time in.*

24. A healthy distance is better than a toxic proximity.

25. Golden rule in relationships: lower the quantity to raise the quality.

26. If you cannot change the context, find a new context.

*B4. Effective communication, and positive perception of shrewdness as a tool for handling relational adversities.*

27. Strong in decision, soft in communication.

28. Trusting others is the best way to unveil their intentions.

29. Ethics without shrewdness are a useless martyrdom.

*B5. The goodness and effectiveness of seducing and changing for others.*

30. If a good relationship is what you are looking for, start attracting others rather than mistreating them.

31. Nature is serious when it comes to attract a partner’s interest.

32. May your absence be heavier than your presence.

33. Become the person your ideal partner would look for.

## Conclusion

This study not only refers to the ancient origins of Ps and As and to the fact that their use has always aimed at improving people’s lives, but it also highlights the principles of the therapeutic value of As. SoO (i.e., the antinomic structure, implicit and explicit) underlies the wit and the surprise effect in As, which in turn underlie their ability to develop a therapeutic reframing. The psychotherapist can specifically choose or devise As in order to arouse aversion toward ISs (which maintain and complicate patients’ problems, impeding the achievement of one’s goals) and to arouse adherence toward ESs (which promote solutions and the achievement of one’s goals). Furthermore, learning how to build therapeutic As involves understanding the difference between psychological opposites and complementaries, i.e., between first-order (apparent) change and second-order (actual) change. In this regard, As with therapeutic effects are an epitome of instructions on how BT and psychological change work.

## Data availability statement

The original contributions presented in the study are included in the article/supplementary material, further inquiries can be directed to the corresponding author.

## Author contributions

BP: Writing – original draft, Writing – review & editing.
